# Platycodin D regulates high glucose-induced ferroptosis of HK-2 cells through glutathione peroxidase 4 (GPX4)

**DOI:** 10.1080/21655979.2022.2045834

**Published:** 2022-02-28

**Authors:** Jinzhong Huang, Gangyi Chen, Jilei Wang, Shibin Liu, Jing Su

**Affiliations:** a Department of Nephrology, The First Affiliated Hospital of Guangzhou University of Traditional Chinese Medicine, Guangzhou City, Guangdong Province, China; b Nephrotic Diagnosis And Treatment Center, The Second Affiliated Hospital of Shandong University of Traditional Chinese Medicine, Jinan City, Shandong Province, China

**Keywords:** Diabetic nephropathy, platycodin D, HK-2 cells, ferroptosis, GPX4

## Abstract

Diabetic nephropathy (DN) is associated with inflammation. Platycodin D (PD) demonstrates anti-inflammatory activity. However, whether PD affects DN remains to be explored. Here, we aimed to discuss the role of PD in DN and its underlying mechanisms. High glucose (HG)-induced HK-2 cells were treated with PD, and cell viability was assessed using the Thiazolyl Blue Tetrazolium Bromide (MTT) assay. Ferroptosis-related factors such as lactate dehydrogenase (LDH) activity, lipid reactive oxygen species (ROS), iron (Fe^2+^) level, GSH level, and malondialdehyde (MDA) level were evaluated. Cell death was evaluated using the TUNEL assay. GPX4 expression was evaluated using Quantitative Real-time PCR (qRT-PCR) and Western blotting analysis. The results indicated that HG increased LDH activity, lipid ROS production, Fe^2+^ levels, and MDA levels and decreased GSH levels, suggesting that the HG condition induced ferroptosis. PD treatment inhibited ferroptosis in HG-induced cells, downregulated ACSL4 and TFR1 expression, and upregulated FTH-1 and SLC7A11 expression. PD reversed the effects of HG condition on cell death. Moreover, GPX4 expression was downregulated in HG-stimulated cells. Furthermore, we substantiated that PD suppressed ferroptosis by modulating GPX4 expression. In conclusion, PD inhibited ferroptosis in HG-induced HK-2 cells by upregulating GPX4 expression, suggesting that PD may be an effective drug for the clinical treatment of DN.

## Graphical abstract

**Figure:**
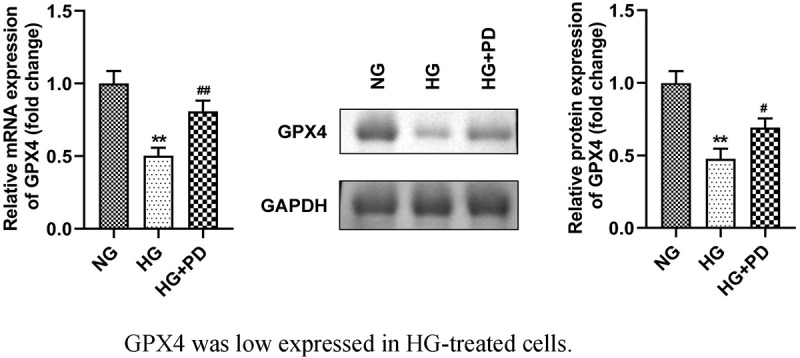

## Introduction

Diabetic nephropathy (DN) is a microvascular complication caused by diabetes and has become a leading cause of end-stage renal disease worldwide [1]. Severe DN can cause kidney failure and even lead to death. DN develops in a quarter of the patients with type 2 diabetes mellitus and is characterized by albuminuria, glomerulopathy, and a decreased glomerular filtration rate [2,3]. However, these indicators are not specific and cannot help diagnose DN. The main risk factors for DN include hyperglycemia, hypertension, hyperlipidemia, heredity, smoking, and diet [4]. Although the current therapeutic strategies for DN have improved, these drugs mainly focus on lowering blood glucose, blood pressure, and proteinuria, and cannot effectively cure DN [5]. Therefore, more effective drugs need to be further investigated.

Renal proximal tubular epithelial cells (PTECs) are involved in the pathogenesis of DN. PTECs are activated by exposure to high glucose (HG) conditions, producing cytokines, thereby inducing tubulointerstitial lesions, including inflammation and fibrosis [6–8]. A previous study has reported that ferroptosis in PTECs is closely related to DN progression [9]. Ferroptosis is a newly discovered form of programmed cell death characterized by iron-dependent lipid peroxidation-induced cell death. It is involved in multiple diseases such as malignancy, cardiovascular disease, and organ injury [10]. Thus, selective inhibition of ferroptosis may be an excellent strategy to treat DN.

Platycodin D (PD), isolated from the dry root of *Platycodon grandiflorum*, is a triterpenoid saponin with multiple pharmacological properties. It has anti-tumor, anti-inflammatory, antiviral, anti-aging, and neuroprotective effects [11–13]. PD attenuates the development of diseases by regulating biological behaviors such as cell growth, apoptosis, metastasis, cell cycle, and autophagy [14,15]. PD has a protective effect against diabetic liver injury [16]. However, the involvement of PD in DN and whether PD affects ferroptosis remains unknown.

Glutathione peroxidase 4 (GPX4) is a key regulator in ferroptosis. It reduces complex hydroperoxides including phospholipid hydroperoxides and cholesterol hydroperoxides to their corresponding counterparts, and thus interrupting the lipid peroxidation chain reaction [17]. Inhibition of GPX4 function causes increased lipid ROS formation and lipid peroxidation, leading to the induction of ferroptosis [18].

Thus, in this study, we aimed to explore the effects of PD on ferroptosis in DN. HK-2 cells were exposed to HG concentration and treated with different concentrations of PD. We hypothesized that PD inhibited ferroptosis by regulating the GPX4. The goal of this study is to provide a basis for the treatment of DN using PD.

## Materials and methods

### Materials

DuIbecco’s modified eagIe’s medium (DMEM) and fetal bovine serum (FBS) were obtained from Gibco (Grand Island, NY, USA). D-glucose, PD (purity ≥ 98% HPLC; Figure 2(a)), Thiazolyl Blue Tetrazolium Bromide (MTT), dimethyl sulfoxide (DMSO), and PBS were purchased from Sigma-Aldrich (St. Louis, MO, USA). Erastin and deferasirox (DFX) were purchased from MedChemExpress (Monmouth Junction, NJ, USA). Small interfering RNA (si)-GPX4 1#, si-GPX4 2#, and its negative control (si-nc) were acquired from GenePharma (Shanghai, China). Lipofectamine 3000, TRIzol, and C11-BODIPY (581/591) were acquired from Invitrogen (Carlsbad, CA, USA). The lactate dehydrogenase (LDH) assay kit was purchased from Jiancheng (Nanjing, China). Reduced and oxidized glutathione (GSH and GSSG, respectively) assay kit, lipid peroxidation malonaldehyde (MDA) assay kit, BCA protein assay kit, PVDF membranes, western blocking buffer, BeyoECL Plus, terminal deoxynucleotidyl transferase biotin-dUTP nick-end labeling (TUNEL) apoptosis assay kit, and BeyoFast SYBR Green one-step qRT-PCR kit were purchased from Beyotime (Shanghai, China). The iron assay kit, primary antibodies (anti-ACSL4, anti-TFR1, anti-FTH-1, anti-SLC7A11, anti-GPX4, and anti-GAPDH), and HRP-conjugated secondary antibody were purchased from Abcam (Cambridge, MA, USA).

### Cells

Human renal PTECs (HK-2) were purchased from ATCC (Manassas, VA, USA). The cells were incubated in DMEM supplemented with 10% FBS in a humidified incubator at 37°C with 5% CO_2_. To establish HG-induced cells, HK-2 cells were treated with 30 mM D-glucose. HK-2 cells exposed to 5.5 mM/L D-glucose served as the normal glucose (NG) group. The cells were harvested after three days. HG- or NG-induced cells were treated with 5 μM erastin or 200 μM DFX to induce or inhibit ferroptosis, respectively.

### PD exposure and cell viability evaluation

HK-2 cells were seeded in 96-well plates. After treatment with PD at concentrations of 0, 1, 2.5, and 5 μM for 24 h, 10 μL MTT was added to the plates. After 4 h, 150 μL DMSO was added to each well to dissolve the formazan crystals. Absorbance was measured at 490 nm using a microplate reader (BioTek, Biotek Winooski, Vermont, USA).

### Cell transfection

Cells in the logarithmic growth phase with 80% confluence were inoculated in 6-well plates. The cells were transfected with si-GPX4 1#, si-GPX4 2#, and si-nc for 48 h using Lipofectamine 3000. Transfection efficiency was estimated using quantitative real-time PCR (qRT-PCR).

### Lactate dehydrogenase (LDH) activity evaluation

The LDH assay kit was used to evaluate the LDH activity. The cells were lysed, and the samples (100 μL) were incubated with matrix buffer (250 μL) and coenzyme I solution (50 μL) at 37°C for 15 min. Then, 2, 4-dinitrophenylhydrazine was added and further incubated for 15 min. After adding the NaOH solution for 3 min, the OD value at 440 nm was measured.

### Lipid reactive oxygen species (ROS) production evaluation

Cells seeded in 6-well plates were incubated with 2 µM C11-BODIPY (581/591) at 37°C for 30 min. The cells were then washed with PBS and resuspended. Lipid ROS levels were assessed using flow cytometry.

### Iron content detection

An iron assay kit was used to detect the iron (Fe^2+^) levels [19]. The cells were washed with cold PBS and centrifuged at 16,000 × *g* for 10 min to obtain the supernatant. Iron buffer (5 μL) was added and incubated with the samples (100 μL) at 37°C for 0.5 h. Then, the samples were incubated with an iron probe (100 μL) at 37°C for 1 h. OD value was immediately measured at 593 nm.

### GSH level evaluation

GSH and GSSG assay kits were used to quantify GSH levels [20]. The cells were washed with PBS and incubated with protein-removing M buffer in the kit at 4°C for 5 min. After centrifugation, the supernatant was incubated with a working buffer at 25°C for 5 min. The samples were then mixed with 50 μl NADPH, and the OD value was detected at 410 nm.

### MDA level evaluation

The lipid peroxidation MDA assay kit was used to determine MDA levels [20]. The cells were homogenized using PBS, and centrifugation was performed at 10,000 × *g* for 10 min to obtain the supernatant. The supernatant (0.1 mL) was mixed with the MDA working buffer (0.2 mL) and heated at 100°C for 15 min. After cooling to 25°C, the OD value was measured at 532 nm.

### Western blotting

Cells were lysed using RIPA lysis buffer on ice, and the protein concentration was evaluated using a BCA protein assay kit. Subsequently, SDS-PAGE was performed, and the proteins were transferred to PVDF membranes. After blocking with western blocking buffer, the membranes were incubated with primary antibodies at 4°C overnight. Then, the membranes were incubated with secondary antibodies at room temperature for 1 h after washing with TBS+Tween-20 (TBST). All bands were imaged using BeyoECL Plus. GAPDH was used as an internal reference.

### TUNEL analysis

The TUNEL apoptosis assay kit was used to quantify cells undergoing apoptosis. The cells were fixed with 4% paraformaldehyde and permeabilized with 0.3% Triton X-100. Then, the cells were incubated with 50 μL Biotin-dUTP buffer at 37°C for 1 h, and TUNEL-positive cells were imaged under a fluorescence microscope (Olympus, Tokyo, Japan).

### qRT-PCR

Total RNA was isolated using TRIzol reagent. After evaluating the purity and integrity, BeyoFast™ SYBR Green one-step qRT-PCR kit was used for reverse transcription and quantitative PCR (qPCR) on an ABI 7500 system (Applied Biosystems). The qPCR conditions were 95°C for 2 min, 95°C for 15s, and 60°C for 30s (40 cycles). The expression of GPX4 was calculated using the 2^−∆∆Ct^ method. *GAPDH* served as a housekeeping gene control.

### Statistical analyses

Data were assessed using GraphPad Prism 7 (La Jolla, CA, USA) and are expressed as the mean ± standard deviation. Differences between two or multiple groups were analyzed using the Student’s *t*-test or one-way analysis of variance. Statistical significance was set at P < 0.05.

## Results

Using a HG-induced HK-2 cell model, we investigated the effect of PD on ferroptosis and the molecular machanism. We analyzed LDH activity, lipid ROS, Fe^2+^ level, GSH level, and MDA level to evaluate the ferroptosis. We found that PD inhibited ferroptosis of HK-2 cells via regulating GPX4. The findings provided a theoretical basis for PD treatment of DN.

### HG mediated ferroptosis in mesangial cells

**Figure 1.**
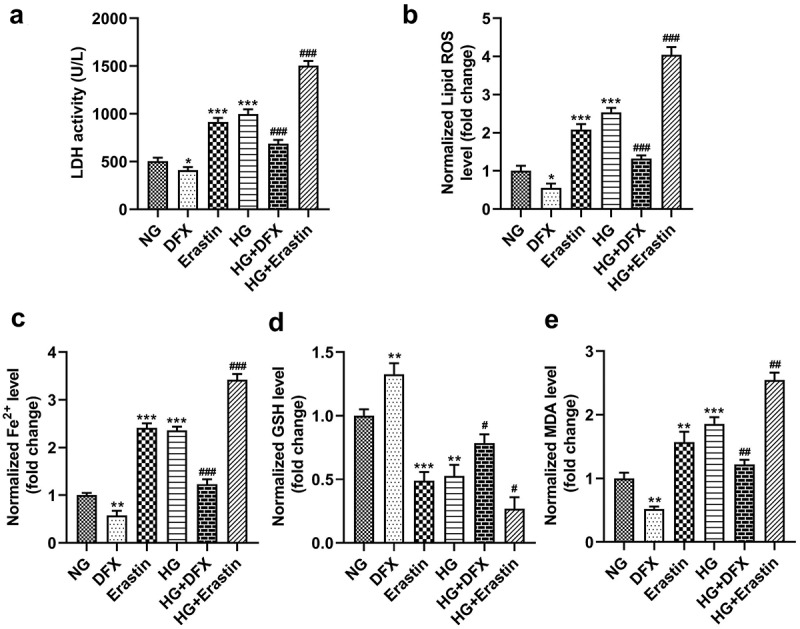
Ferroptosis in mesangial cells was mediated by high glucose (HG). (a) LDH activity. (b) Lipid ROS production. (c) Fe^2+^ levels. (d) GSH levels. (e) MDA levels. *P < 0.05, **P < 0.01, and ***P < 0.001 vs. NG group. #P < 0.05, ##P < 0.01, and ###P < 0.001 vs. HG group.

HK-2 cells were incubated in NG or HG conditions, and both were treated with DFX and erastin. The data showed that HG significantly promoted LDH activity and lipid ROS production and increased Fe^2+^ content and MDA levels but significantly reduced GSH levels. DFX treatment markedly suppressed LDH activity, lipid ROS production, Fe^2+^ levels, and MDA levels but markedly elevated GSH levels in NG- and HG-induced cells. By contrast, erastin markedly upregulated LDH activity, lipid ROS production, Fe^2+^ levels, and MDA levels but decreased GSH levels (Figure 1(a–e)).

**Figure 2.**
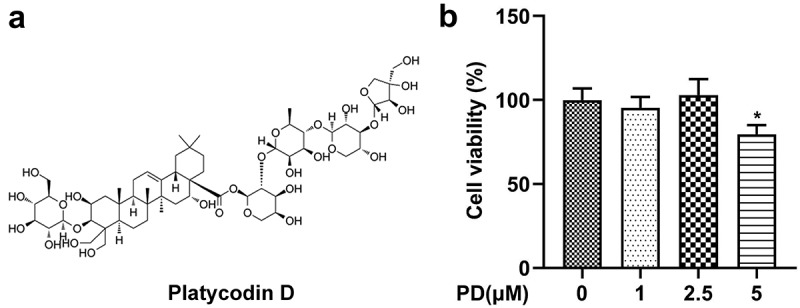
Effect of platycodin D (PD) on cell viability. (a) The chemical structure of PD. (b) Cell viability was analyzed following treatment with 0, 1, 2.5, and 5 μM PD. *P < 0.05.

### Effect of PD on cell viability

After cells treating with PD, cell viability was assessed. The results showed that cell viability was not affected by 0, 1, and 2.5 μM PD but was markedly suppressed by 5 μM PD (Figure 2(b)).

### PD inhibited HG-induced ferroptosis

**Figure 3.**
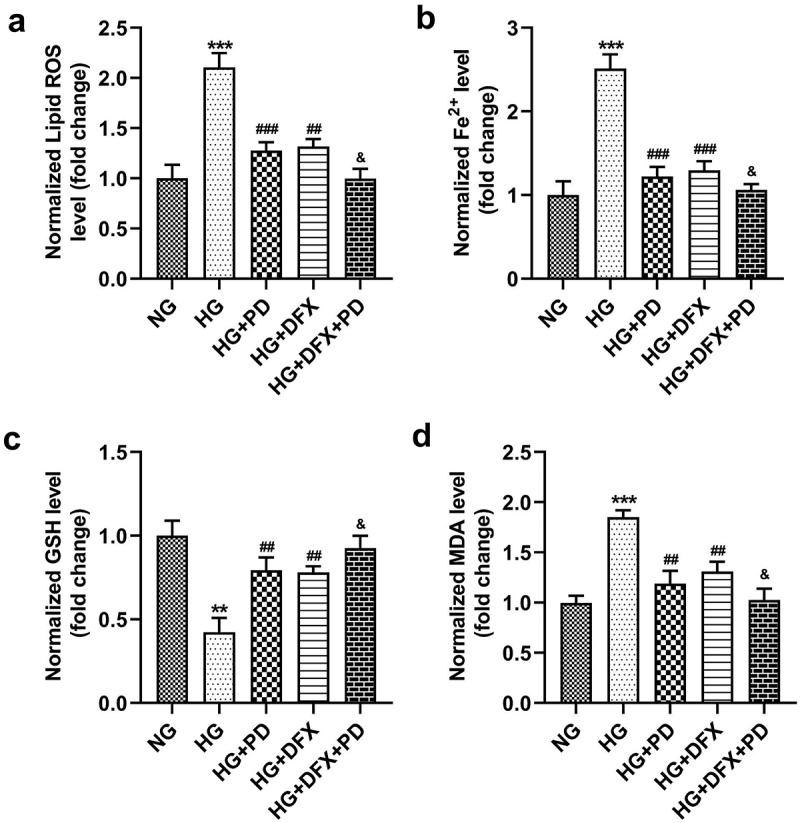
PD inhibited HG-induced ferroptosis. (a) Lipid ROS production. (b) Iron levels. (c) GSH levels. (d) MDA levels. **P < 0.01 and ***P < 0.001 vs. NG group. ##P < 0.01 and ###P < 0.001 vs. HG group. &P < 0.05 vs. HG + PD group.

**Figure 4.**
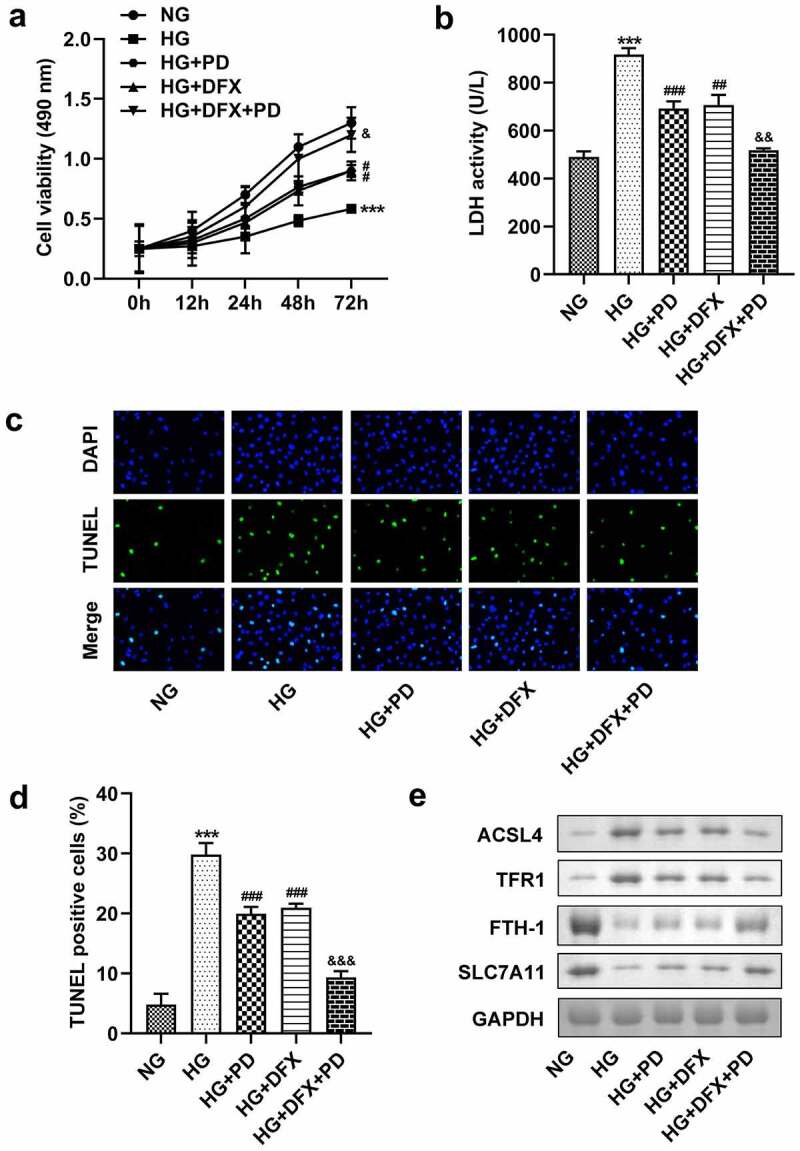
PD suppressed the death of HK-2 cells. (a) Cell viability. (b) LDH activity. (c–d) Cell death. (e) ACSL4, TFR1, FTH-1, and SLC7A11 levels. **P < 0.01 and ***P < 0.001 vs. NG group. ##P < 0.01 and ###P < 0.001 vs. HG group. &P < 0.05, &&P < 0.01, and &&&P < 0.001 vs. HG + PD group.

To explore whether PD affected ferroptosis, HG-treated cells were exposed to 2.5 μM PD. PD decreased lipid ROS production, Fe^2+^ levels, and MDA levels and increased GSH levels in HG-treated cells, whereas DFX further exacerbated the effect of PD (Figure 3(a–d)). Additionally, PD increased the viability of HK-2 cells (Figure 4(a)). PD significantly attenuated the HG-induced enhancement of LDH activity, and DFX markedly suppressed LDH activity in PD-treated cells (Figure 4(b)). Furthermore, PD decreased the number of HG-induced, TUNEL-positive cells, and DFX further decreased the number of dead cells (Figure 4(c,d)). ACSL4 and TFR1 levels were elevated, whereas FTH-1 and SLC7A11 levels decreased after HG addition. Thus, PD abrogated the effects of HG, and DFX further enhanced the effects of PD (Figure 4(e)).

### GPX4 expression was low in HG-treated cells

**Figure 5.**
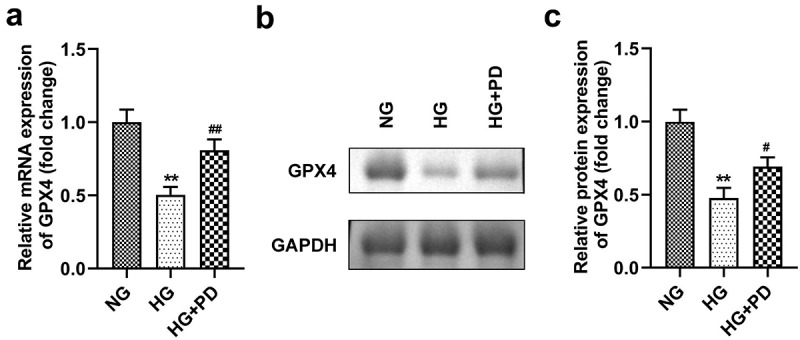
GPX4 expression was low in HG-treated cells. (a) The mRNA expression of GPX4. (b) Western blotting analysis was used to determine GPX4 expression. (c) Quantification of GPX4 expression. **P < 0.01, ***P < 0.001, #P < 0.05, and ##P < 0.01.

As illustrated in Figure 5(a), GPX4 expression was significantly downregulated in cells exposed to HG, which was alleviated by PD. Moreover, HG markedly reduced the GPX4 protein levels, whereas PD perceptibly rescued this reduction (Figure 5(b,c)).

### PD inhibited ferroptosis by regulating GPX4

**Figure 6.**
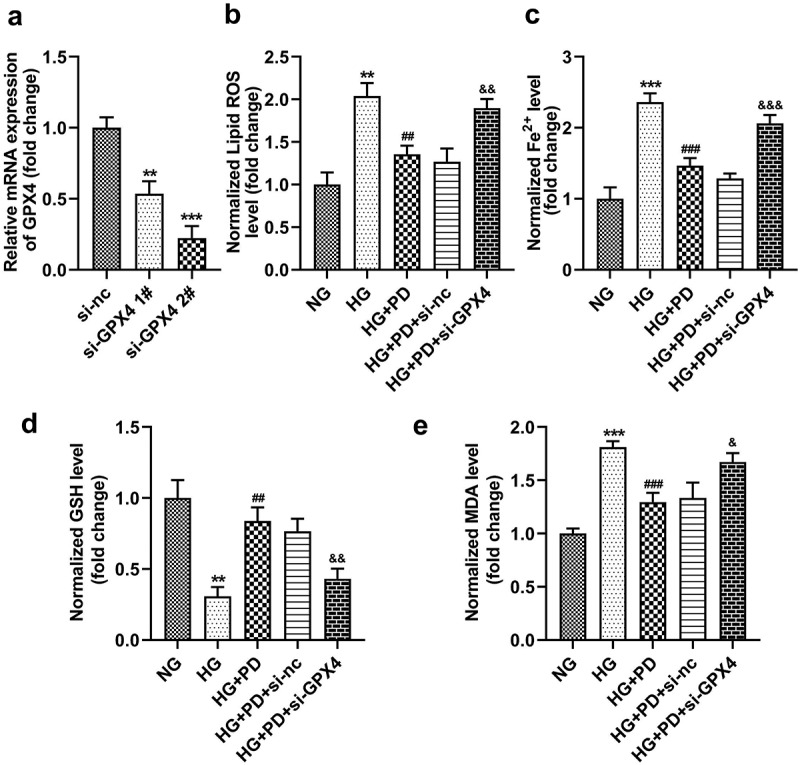
GPX4 knockdown promoted HG-induced ferroptosis. (a) GPX4 was measured after transfection. (b) Lipid ROS production. (c) Iron levels. (d) GSH levels. (e) MDA levels. **P < 0.01 and ***P < 0.001 vs. si-nc or NG group. ##P < 0.01 and ###P < 0.001 vs. HG group. &P < 0.05, &&P < 0.01, and &&&P < 0.001 vs. HG + PD + si-nc group.

**Figure 7.**
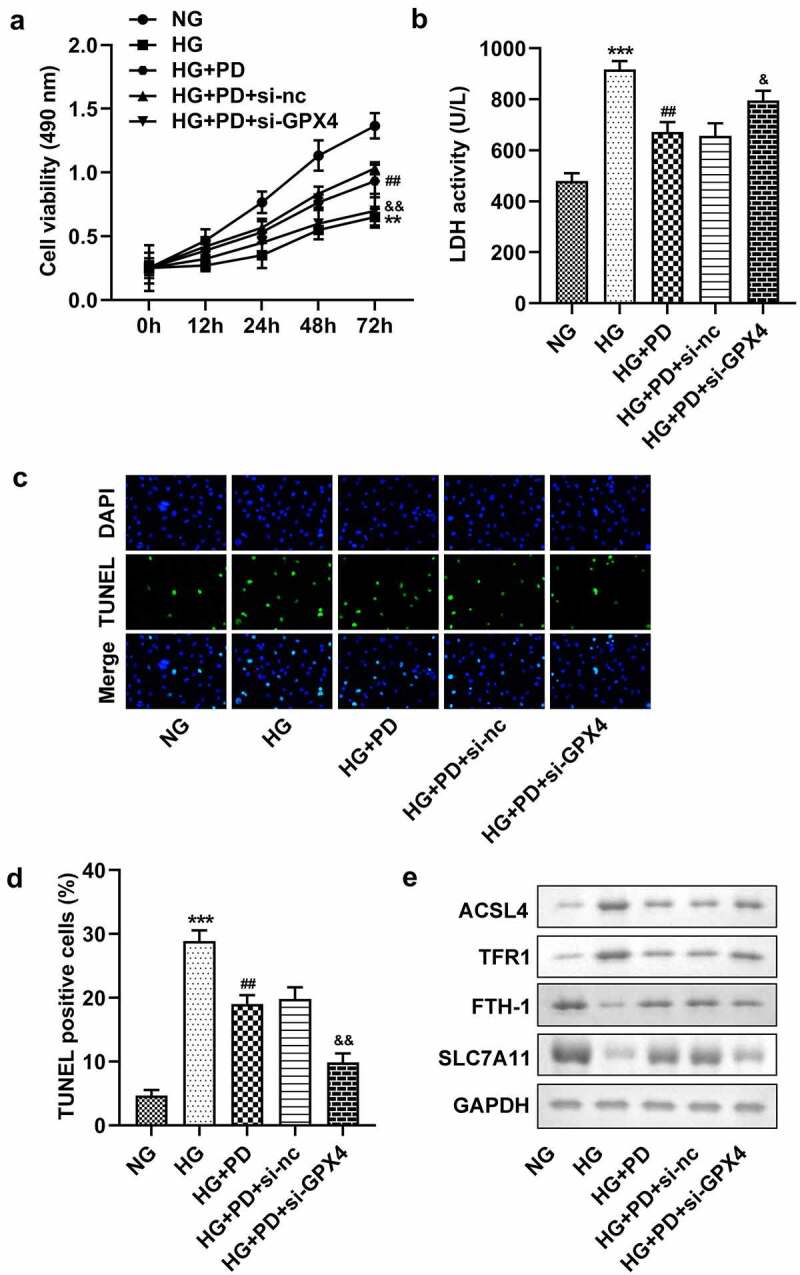
GPX4 knockdown promoted HG-induced cell death. (a) Cell viability. (b) LDH activity. (c–d) Cell death. (e) ACSL4, TFR1, FTH-1, and SLC7A11 levels. **P < 0.01 and ***P < 0.001 vs. si-nc or NG group. ##P < 0.01 vs. HG group. &P < 0.05, &&P < 0.01 vs. HG + PD + si-nc group.

Following transfection, the levels of GPX4 markedly decreased in the si-GPX4 1# and si-GPX4 2# groups (Figure 6(a)). GPX4 silencing significantly reversed the effect of PD on ferroptosis-related indicators, including lipid ROS production and Fe^2+^, GSH, and MDA levels (Figure 6(b–e)). It also significantly suppressed the viability of HK-2 cells (Figure 7(a)). Moreover, the LDH activity of HK-2 cells transfected with si-GPX4 significantly increased (Figure 7(b)). Furthermore, GPX4 silencing significantly abolished the effect of PD on cell death in HG-induced cells (Figure 7(c,d)). PD notably downregulated ACSL4 and TFR1 but upregulated FTH-1 and SLC7A11 in HG-induced cells, whereas GPX4 knockdown abrogated the effect induced by PD (Figure 7(e)).

## Discussion

Here, we investigated whether PD was involved in the progression of DN. Moreover, the mechanism of action of PD on ferroptosis was investigated. We found that PD suppressed ferroptosis in HG-induced cells by regulating GPX4 expression.

Ferroptosis regulates cell death in many diseases, including neurodegenerative disorders, ischemia-reperfusion injury, malignancy, and kidney diseases [21,22]. Growing evidence has revealed that ferroptosis is associated with acute kidney injury, chronic kidney disease, and tubular necrosis [23]. Ferroptosis changes cell morphology, resulting in smaller mitochondria, increased mitochondrial membrane density, and decreased mitochondrial crest, which are different from the characteristics of apoptosis, autophagy, and necrosis. The iron, lipids, and amino acids metabolic pathways are involved in the regulation of ferroptosis [24]. GSH consumption decreases GPX4 activity, resulting in lipid peroxides not being metabolized [22]. Iron levels are a main indicator of ferroptosis; iron oxidizes lipids, leading to excess ROS production [25]. DFX is an iron chelator that can decrease iron content and inhibit the production of intracellular lipid reactive oxygen, attenuating proximal tubular injury and glomerulosclerosis [25,26]. Erastin is a ferroptosis inducer that forms ineffective antioxidant defenses by mediating the amino acid antiporter, system XC^−^ [27]. In this study, we found that HG increased LDH activity, lipid ROS production, Fe^2+^ levels, and MDA levels and decreased GSH levels, suggesting that HG levels induced ferroptosis. These data are consistent with those of previous studies [28,29]. Moreover, DFX treatment suppressed ferroptosis, whereas erastin facilitated ferroptosis. The findings suggested that HG induced ferroptosis of HK-2 cells.

*P. grandiflorum* is a traditional Chinese herb commonly used to treat respiratory diseases. Its extract increases insulin-induced glucose uptake, suggesting that this herb could contribute to the treatment of diabetes [30]. PD regulates hepatic lipogenesis in HG-induced adipocytes and alleviates liver injury in mice with diabetes [16,31]. Additionally, PD can relieve renal toxicity and protect the kidneys [32]. Growing evidence has revealed that PD can be beneficial in the treatment of in cancers, osteoporosis, and neurological diseases [11,33,34]. However, it was unknown whether PD played a role in DN. This study established that PD inhibited ferroptosis in HK-2 cells, whereas DFX treatment abrogated ferroptosis. These data suggest that PD might attenuates DN progression by suppressing ferroptosis.

GPX4 decreases phospholipid hydroperoxide and acts as a regulator of ferroptosis by erastin [35]. GPX4 levels decreased in the kidney specimens of patients with diabetes and were associated with ferroptosis in the kidney tubular cells [36]. Moreover, GPX4 is downregulated in DN mice and has an anti-lipid ROS production effect that suppresses ferroptosis [9]. Our study showed that GPX4 expression was reduced in HG-stimulated cells and that PD induced upregulation of GPX4 expression. Moreover, si-GPX4 reversed the effects of PD. These findings suggest that PD inhibits ferroptosis in HG-induced cells by regulating GPX4 expression.

The novelty of this study is that PD can alleviate ferroptosis in HK-2 cells for the first time, suggesting that PD may help treat DN. However, more studies are needed to apply PD to clinical treatment of DN. The main limitation of this study is that we only analyzed the effect of PD on HK-2 cells. Additionally, the effect of PD on other biological behaviors of HK-2 cells are also unclear. We will explore the role of PD in other cells and the effect of PD on other biological functions in our future work.

## Conclusion

In short, ferroptosis in HK-2 cells was induced by HG and was suppressed by PD. GPX4 expression was downregulated in HG-induced cells and was upregulated by PD. Thus, PD suppressed ferroptosis of HK-2 cells by upregulating GPX4 expression, suggesting that PD might be an effective drug for DN therapy.

## Data Availability

The datasets used during the current study are available from the corresponding author on reasonable request.
